# Clean air for a healthier Western Pacific region

**DOI:** 10.1016/j.lanwpc.2026.101996

**Published:** 2026-09-30

**Authors:** The Lancet Regional Health – Western Pacific

Clean air is not a privilege, but a prerequisite for health and human rights. Recognising this, the UN General Assembly designated Sept 7 as International Day of Clean Air for Blue Skies, calling attention to the need for stronger action on air pollution.

In the Western Pacific region, this call is particularly urgent. The region shares with southeast Asia the world's greatest burden of premature deaths attributed to air pollution. In May, 2026, the WHO Regional Office for the Western Pacific published its first comprehensive overview of air-quality standards across the region. Although half of countries have established standards for at least one pollutant, none have national standards fully aligned with WHO global air-quality guidelines. An estimated 1.1 million deaths could be prevented each year if countries across the Western Pacific region achieved the WHO guideline annual mean concentration for particulate matter (PM_2.5_) of 5 μg/m^3^.

Setting standards is one part of the solution; a major challenge is that air pollution sources differ substantially within and between countries across the Western Pacific region. Within China, a source attribution study suggested that industrial emissions contribute significantly to pollution-related health burdens in developed coastal areas, whereas residential emissions predominate in the colder northeast, and natural sources contribute more in parts of the less developed northwest. Moreover, particulate matter from different sources might not pose equivalent health risks. A multicountry study including Australia, South Korea, and New Zealand reported that traffic-sourced PM_2.5_ was more strongly associated with cancer mortality than total PM_2.5_, despite accounting for a small portion of overall concentrations. The transboundary nature of air pollution adds another layer of complexity, as air pollution respects no borders. Although countries can strengthen domestic policies, communities living downwind may remain vulnerable if upwind sources are not adequately addressed. Fine particulate pollution could be generated by a combination of domestic, transboundary, and other external sources, whose relative contributions vary across seasons and pollution episodes. Such uncertainty in source attribution can obscure political accountability and complicate decisions about where responsibility for mitigation should lie.

There is therefore no one-size-fits-all route to cleaner air in the Western Pacific. Stronger air-quality standards should provide a common health goal, but how countries reach that goal must reflect where their pollution comes from and the resources available to address it. Governments should prioritise the sources responsible for the greatest local health burden and invest in monitoring systems capable of identifying them. In lower-resource settings where technical capacity is limited, regional partnerships can support monitoring infrastructure, comparable data collection, and source attribution. For pollution that crosses borders, domestic action must be complemented by regional cooperation. Neighbouring jurisdictions need shared emissions information and coordinated mitigation strategies that link pollution measured downwind with action at its source. Health authorities should also be integral to these coordinated efforts. Linking air-quality monitoring with health surveillance and health impact assessment can help identify populations at greatest risk, support coordinated public communication during pollution episodes and ensure that mitigation strategies are evaluated against measurable health outcomes.

The benefits of clean air can be extended beyond air quality. Many sources of ambient air pollution, particularly fossil fuel combustion, also produce greenhouse-gas emissions. Reducing the use of polluting fuels can simultaneously improve air quality and reduce emissions that contribute to climate change. This connection is particularly important in a region already vulnerable to climate-related health threats, including extreme heat, wildfires, and other environmental hazards. Clean air policy should therefore not sit in isolation from broader efforts to build healthier and more resilient communities.

On this International Day of Clean Air for Blue Skies on Sept 7, the message for the Western Pacific is clear: cleaner air will depend on combining a common health ambition with locally appropriate action and regional responsibility. No country can achieve clean air alone, but every country can contribute to cleaner air for all.

